# Book Review: Positive Psychology in Second and Foreign Language Education

**DOI:** 10.3389/fpsyg.2021.812299

**Published:** 2021-12-02

**Authors:** Xue Wang, Erchen Yue

**Affiliations:** ^1^Department of Foreign Languages, Central China Normal University, Wuhan, China; ^2^Basic Teaching Department, Information Engineering University, Zhengzhou, China

**Keywords:** positive psychology, second and foreign language, language education, EFL (english as a foreign language), psychology

The flowering of Positive Psychology (PP) has revolutionized not only general education but also Second Language Acquisition (SLA) as well as second/foreign language education (Gabryś-Barker and Gałajda, 2016; MacIntyre et al., 2016; Wang et al., 2021). Therefore, any academic endeavor seems to fill the gap in research. Katarzyna Budzińska and Olga Majchrzak's edited volume, titled *Positive Psychology in Second and Foreign Language Education*, delineates how resources drawn from PP can enlighten teachers, learners, and researchers. It also adds more innovative topics to the field such as affectivity, positive emotions, a positive L2 self-system, and positive language education.

The book is divided into three sections, namely theoretical, empirical, and applied. The theoretical section includes four chapters. Chapter 1 commences the theoretical underpinnings of PP by highlighting the applications of PP in SLA, defining its key terminologies, and cautioning the readers about “the risk of applying positive psychology principles in an oversimplified manner” (p. 2). Taking an ecological perspective into account as an innovative springboard, Chapter 2 introduces a benchmark for understanding language teacher well-being, incorporating teachers' professional, personal, emotional lives into their careers. Chapters 3 and 4 focus on the significance of PP for students. The author in Chapter 3 advocates a cordial teacher-student and student-student relationship in the international higher education context, bringing to the fore the “empathic teachers who are willing to question their own assumptions and to assume their roles and responsibilities for nurturing and nourishing teacher-student and student-student relationships” (p. 43). The last chapter in this section synthesizes the results of some empirical papers on gender differences in foreign language enjoyment (FLE). The authors highly “advised not to treat gender as a determinant of either high or low FLE but to focus on other factors that may boost FLE in all FL learners, regardless of gender” (p. 61).

The second section of the book deals with the empirical side of PP that covers five chapters. Chapter 5, drawing on the biographical narrative and metaphor gleaned from 78 freshmen students of pedagogy, integrates PP, positive L2 self, as well as L2 motivation. The chapter reports that motivational variables can have a bearing on the positive L2 self-system. Chapter 6 probes into the interplay between different dimensions of willingness to communicate (WTC), engagement, and communicative behavior in task performance in the Polish context. The author concludes that the “correlation between communicative behavior and questionnaire scores were insignificant” (p. 103). Besides, the chapter reports that “comparisons of means for WTC, engagement, word count and turns failed to capture an interplay of cognitive, affective, motivational and behavioral variables involved in task performance” (p. 118). Chapter 7, enlightened by Seligman's (2011) PERMA model, takes into account both learners' and the teachers' perspectives by identifying characteristics of the institution's policy that culminates in a framework of Positive Language Education (PLE) as one of the pillars of PP. The author cogently argues that “It is vital that the institution also embodies and communicates the principles of PLE in its structures, policies, and organizational culture” (p. 143). Set in the Polish context, Chapter 8, utilizing a three-stage longitudinal duoethnographic design, focuses on the construction of trainee teachers' identity with respect to different dimensions of PP. The chapter recapitulates that “the application of duoethnographic dialogues in teacher preparation programmes may offer a way of capturing its emergence in order to learn both about teacher identity and from teacher identity” (p. 146). The last chapter in this section examines the concept of teaching as a profession and argues that teaching training programs need to enhance teachers' effectiveness and motivation and help them manage their resources. The authors recommend that “positive psychology and neurodidactics are necessary for a sustainable and successful teaching career” (p. 171).

The third section, applied, encompasses two chapters. Developing an effective teacher training program by drawing on positive affectivity for pre-service teachers is the focus of Chapter 10. The author contends that “it is the teacher that takes responsibility for his or her classes and individual learners in communicating and interacting in the process of language instruction” (p. 204). The last chapter, using biographical narrative and metaphor, aims to enhance “learners' reflectiveness, self-cognition, their ability to perceive the world and their own lives from different perspectives” (p. 226), by assisting students to become cognizant of essential values and meaning in their lives.

We are fully confident that this compendium can serve as a springboard not only for teachers, learners, and researchers but also for policymakers and stakeholders who can nourish and nurture the well-being of teachers and learners in EFL/ESL contexts. This volume is adorable in that it reconciles theory and practice since half of the book is dedicated to data-driven chapters and the other half goes to the underlying theoretical postulations about the three pillars (i.e., positive emotions and feelings, positive characteristics and personality traits, and positive institutions) of PP. We are certain that this book will shed more light on the advancement of positive PP in SLA inasmuch as the fact that this volume adds more innovative topics such as “the role of positive psychology in international higher education, a framework for understanding language teacher well-being from an ecological perspective, or positive institutional policies in language education contexts” (p. vi). However, had the editors invited authors from other contexts, not just the Polish EFL context, the volume could have been of interest to a wider range of readership.

## Author Contributions

Both authors listed have made a substantial, direct, and intellectual contribution to the work and approved it for publication.

## Conflict of Interest

The authors declare that the research was conducted in the absence of any commercial or financial relationships that could be construed as a potential conflict of interest.

## Publisher's Note

All claims expressed in this article are solely those of the authors and do not necessarily represent those of their affiliated organizations, or those of the publisher, the editors and the reviewers. Any product that may be evaluated in this article, or claim that may be made by its manufacturer, is not guaranteed or endorsed by the publisher.

## References

[B1] Gabryś-Barker D. Gałajda D. (2016). Positive Psychology Perspectives on Foreign Language Learning and Teaching. Cham: Springer.

[B2] MacIntyre P. D. Gregersen T. Mercer S. (2016). Positive Psychology in SLA. Bristol: Multilingual Matters.

[B3] Seligman M. E. P. (2011). Flourish: A Visionary New Understanding of Happiness and Well-Being. New York, NY: Free Press.

[B4] Wang Y. L Derakhshan A. Zhang L. J. (2021). Researching and practicing positive psychology in second/foreign language learning and teaching: the past, current status and future directions. Front. Psychol. 12, 1–10. 10.3389/fpsyg.2021.731721 34489835 PMC8417049

